# Evaluation of urinary acidification in children: Clinical utility

**DOI:** 10.3389/fped.2022.1051481

**Published:** 2022-10-31

**Authors:** Lucas Díaz-Anadón, Leire Cardo, Fernando Santos, Helena Gil-Peña

**Affiliations:** ^1^ Division of Pediatric Nephrology, Department of Pediatrics, Hospital Universitario Central de Asturias, Oviedo, Asturias, Spain; ^2^ Department of Medicine, Faculty of Medicine, University of Oviedo, Oviedo, Asturias, Spain; ^3^ Clinical Biochemistry Department, Laboratory of Medicine, Hospital Universitario Central de Asturias, Oviedo, Asturias, Spain; ^4^ Section of Pediatrics, Instituto de Investigación Sanitaria del Principado de Asturias (ISPA), Oviedo, Asturias, Spain

**Keywords:** urinary acidification, urine pH, urinary ammonium, spot urine sample, hyperchloremic metabolic acidosis, incomplete distal renal tubular acidosis

## Abstract

The kidney plays a fundamental role in acid-base homeostasis by reabsorbing the filtered bicarbonate and by generating new bicarbonate, to replace that consumed in the buffering of non-volatile acids, a process that leads to the acidification of urine and the excretion of ammonium (NH_4_^+^). Therefore, urine pH (UpH) and urinary NH_4_^+^ (UNH_4_^+^) are valuable parameters to assess urinary acidification. The adaptation of automated plasma NH_4_^+^ quantification methods to measure UNH_4_^+^ has proven to be an accurate and feasible technique, with diverse potential indications in clinical practice. Recently, reference values for spot urine NH_4_^+^/creatinine ratio in children have been published. UpH and UNH_4_^+^, aside from their classical application in the study of metabolic acidosis, have shown to be useful in the identification of incomplete distal renal tubular acidosis (dRTA), an acidification disorder, without overt metabolic acidosis, extensively described in adults, and barely known in children, in whom it has been found to be associated to hypocitraturia, congenital kidney abnormalities and growth impairment. In addition, a low UNH_4_^+^ in chronic kidney disease (CKD) is a risk factor for glomerular filtration decay and mortality in adults, even in the absence of overt metabolic acidosis. We here emphasize on the need of measuring UpH and UNH_4_^+^ in pediatric population, establishing reference values, as well as exploring their application in metabolic acidosis, CKD and disorders associated with incomplete dRTA, including growth retardation of unknown cause.

## Introduction: physiology and technical aspects

### The role of urinary acidification in maintaining acid-base balance

The kidney plays a fundamental role in acid-base homeostasis by regulating plasma bicarbonate (HCO_3_^−^) concentration, which constitutes the metabolic component of acid-base balance. This process is made up of two parts: the reabsorption of filtered HCO_3_^−^ and the generation of new HCO_3_^−^, to replace that consumed by endogenous or exogenous acids (1). HCO_3_^−^ is freely filtered at the glomerulus and then almost completely reabsorbed, making urine virtually free of HCO_3_^−^ under normal conditions. This reabsorption takes place mostly (approximately 80%) in the proximal tubule, the distal segments of the nephron also playing a significant role in this process (1).

The production of new HCO_3_^−^ occurs by excreting acids into urine (urinary acidification), since the addition of an alkali and the loss of acid are essentially equivalent in physiologic systems. The capacity of the nephron to excrete acids as free protons (H^+^) is limited (urinary H^+^ concentration is <0.1 mmol/L even at a urine pH of 4.5) (1). Instead, acid excretion occurs in the distal nephron by two means: excretion of titratable acids and excretion of ammonium (NH_4_^+^), so net acid excretion (NAE) in the urine is calculated as the sum of those components minus urinary HCO_3_^−^ (which is negligible in fasting normal conditions).

Titratable acids refer to the excretion of H^+^ bound to urinary buffers (mainly dibasic/monobasic phosphate) (2) and, in normal conditions, it represents one-third to one-half of NAE. The rest of NAE corresponds to NH_4_^+^ generation and excretion. NH_4_^+^ is synthesized in the proximal tubule by the catabolism of glutamine, generating HCO_3_^−^ in the process (1). In order to have a net gain of HCO_3_^−^, the NH_4_^+^ produced in the kidney must be excreted in the urine, allowing for the reabsorption of HCO_3_^−^ into the bloodstream. If the NH_4_^+^ is not excreted, it returns to the liver, where it is metabolized to urea, consuming an equimolar quantity of HCO_3_^−^ (1, 3). NH_4_^+^ excretion has a greater ability to increase under acid load conditions than titratable acid excretion −for example, the amount of phosphate in the urine is not significantly increased in chronic metabolic acidosis, whereas urinary NH_4_^+^ (UNH_4_^+^) increases several fold− (4) and, therefore, constitutes the most important mechanism of urinary acidification in response to a noxa or stressing condition.

Due to the importance of urinary acidification in maintaining acid-base balance, its evaluation is mandatory in some clinical situations. Although urine pH (UpH) is the simplest, most available parameter to assess urinary acidification, it does not always faithfully reflect NAE, since it merely indicates the maximum concentration of H^+^ that can be achieved in concrete circumstances. For instance, in cases of chronic metabolic acidosis, when renal ammoniagenesis is increased several fold and H^+^ ions are buffered by NH_3_, a relatively high UpH can be observed even when the urinary acidification capacity is preserved (3, 5, 6). Conversely, an appropriately low urine pH (<5.5) can occur when NH_4_^+^ excretion is compromised in cases such as hypoaldosteronism (6, 7). For these reasons, in order to perform a correct evaluation of urinary acidification, UpH should be taken in consideration simultaneously with UNH_4_^+^.

Nevertheless, due to historical technical difficulties, direct UNH_4_^+^ measurement is not usually performed in clinical laboratories as a routine test, and it is estimated by indirect methods, such as the urinary anion gap and the urinary osmolal gap (8, 9), which do not always correlate reliably with UNH_4_^+^ (10–15). To solve this problem, automated plasma NH_4_^+^ quantification methods, available in most laboratories, have been adapted and evaluated to analyze UNH_4_^+^, proving to be an accurate and simple technique for direct UNH_4_^+^ measurement (11, 16–18), also avoiding the inaccuracies resulting from urinary anion and osmolal gaps. These methods allow the use of direct UNH_4_^+^ quantification in clinical practice as a routine test to assess urinary acidification, reducing the number of tests, analysis time and sample volume, an issue of particular importance in children.

### UpH measurement

A potentiometric pH meter is the gold standard method for UpH measurement. In clinical practice, dipsticks are often used, which are much more available but less accurate, providing a value only up to the nearest 0.5 unit interval and being prone to perception bias when electronic readers are not used. Although dipsticks are useful in most situations, differences between dipstick and pH-meter readings can be as high as 0.4–0.5 units (19–21), even with electronic readers (22, 23). These differences might be clinically significant and thus lead to wrong clinical decisions in specific contexts (22).

UpH may be analyzed in freshly collected urine, or in a sample collected under mineral oil to minimize CO_2_ diffusion when measurement is delayed (especially when pH values ≥6.0 are expected) (24). However, collection under mineral oil can be omitted if the sample is stored at 4 °C in a regular disposable plastic syringe (capping the drawing needle and avoiding air bubbles) and the measurement is performed within 24 hours (25).

As UpH is a measure of H^+^ concentration, its value is dependent on urine concentration status. An extremely diluted urine may provide a falsely elevated UpH reading even when H^+^ excretion is normal, so an overnight thirst period is recommended before collecting the sample. It should also be taken in consideration that UpH follows a circadian rhythm, decreasing during the night, reaching its minimum before dawn and rising after common western meals (26–28). A low spontaneous UpH measured in a fasting spot morning urine is considered to rule out an acidification disorder without the need for more specific tests. In fact, fasting morning UpH has shown a better correlation with nadir UpH after an ammonium chloride (NH_4_Cl) load −the gold standard for the evaluation of urinary acidification− than 24-hour UpH (29). There is no consensus on the type of sample: the first vs. the second (usually after a period of 1–2 h after awakening) fasting morning urine sample. Chafe and Gault (30) reported that the first morning urine pH might be a better predictor of urinary acidification after an NH_4_Cl load than the second fasting urine. The morning rise of UpH, even in the absence of food or drink, has been well reported as a part of its circadian rhythm (26, 31, 32) and might account for this result. Consequently, the first morning urine, which is easier to collect and avoids the problems of a prolonged fasting period, could be the best option to evaluate UpH in continent children. Special considerations should be taken into account in cases of nocturnal enuresis and in infants who feed at night.

### UNH_4_^+^ measurement

In the absence of metabolic acidosis and, outside the context of functional tests, UNH_4_^+^ is usually measured in 24-hour urine. A review on the available data on daily NH_4_^+^ excretion in adults under normal and various pathological conditions has recently been published (15). In children, there are very few data. Manz et al. (33) measured daily NAE in healthy children between 3 and 18 years old, but UNH_4_^+^ values were not reported.

24-hour urine collection is difficult to perform, requiring preservatives to avoid increases in UNH_4_^+^ until the time of analysis and bladder catheterization in young children and infants. Some studies have used spot urine samples in adults (34, 35), which are easier to collect, but UNH_4_^+^ concentration may not represent faithfully NH_4_^+^ excretion. To minimize the effect of variations in urine flow and concentration, a better parameter is the urine NH_4_^+^/creatinine ratio measured in the same sample.

Renal NH_4_^+^ excretion shows also a circadian rhythm (36) and increases following protein intake (37), so the best time for sample collection may be the first fasting morning urine. In fact, our group recently published urine NH_4_^+^/creatinine ratio reference values for children over 5 years old in spot morning urine, ranging between 776 and 8217 µmol/mmol (38). This may clear the path for applying direct spot UNH_4_^+^ measurement in daily clinical practice in children.

Traditionally, individual samples for UNH_4_^+^ quantification were collected in paraffin and shipped to the laboratory on ice (39), but we showed no significant differences between samples collected with or without paraffin (17), so samples can be collected in tubes without additives and sent to the laboratory without further preparation. UNH_4_^+^ concentrations have proven also to be stable up to 48 h at room temperature, up to 9 days at 4 °C and −20 °C, and for at least 2 years when stored at −80 °C (18). However, in order to avoid contamination and bacterial growth, they should be centrifuged, separated and then analyzed as soon as possible or frozen if the analysis is delayed.

Taking all the above into consideration, the first fasting morning urine, in our opinion, is the best sample to evaluate UpH and UNH_4_^+^/creatinine ratio simultaneously.

## Uph and UNH_4_^+^ measurements in metabolic acidosis

The main indication for evaluating urinary acidification in children is during the assessment and diagnosis of normal anion gap (hyperchloremic) metabolic acidosis, which can result, among other causes, from the loss of HCO_3_^−^ from the gastrointestinal tract or kidney (proximal renal tubular acidosis –pRTA–), the addition of hydrochloric acid (HCl) or substances metabolized to HCl, or an impaired net renal acid excretion, as happens in distal renal tubular acidosis –dRTA– or in chronic kidney disease (CKD), when glomerular filtration rate (GFR) is significantly reduced (usually <20–25 ml/min/1.73 m^2^) (40).

The extrarenal (digestive) causes of hyperchloremic metabolic acidosis are the most common. In this context, when kidney function is preserved, UpH should be low and UNH_4_^+^ should rise. The increase in UNH_4_^+^ excretion is modest initially, but is maximized after 3–5 days if the stimulus persists (1, 3, 6). When more frequent extrarenal causes and CKD are excluded, an assessment of urinary acidification is indicated (40, 41). In these situations, an inappropriately high UpH with low UNH_4_^+^, coexisting with metabolic acidosis, indicate an impaired distal urinary acidification (42). If distal acidification is preserved, efforts should be directed to evaluate renal HCO_3_^−^ wasting (40). Usually, it is not necessary to measure UpH and UNH_4_^+^ in most cases, since the underlying disorder is usually identifiable with a comprehensive history, physical examination and basic laboratory tests. However, the determination of UpH and UNH_4_^+^ in single spot urine can be a useful and simple tool in the assessment of normal anion gap metabolic acidosis, especially when the cause is not clear or an underlying kidney pathology is suspected (40, 41).

Another less frequent indication for UpH and UNH_4_^+^ measurement in pediatrics is during the realization of functional urinary acidification tests for the confirmatory diagnosis of dRTA, such as the oral NH_4_Cl load (43) and the furosemide + fludrocortisone test (29, 44, 45). In healthy subjects, UpH should decrease below 5.3 in both tests (although this precise value is disputed) and UNH_4_^+^ should rise up to 57 ± 14 (mean ± SD) µEq/min/1.73 m^2^ in infants aged 1–16 months and 80 ± 12 µEq/min/1.73 m^2^ in children aged 7–12 years (45) in the oral NH_4_Cl load.

However, dRTA in children is mostly primary, caused by genetic alterations (41, 42, 46, 47) and the increasing availability of genetic testing has partly relegated functional tests in this context.

## Incomplete distal renal tubular acidosis (dRTA)

Incomplete dRTA is a disorder defined by an inability to maximally acidify urine in the absence of spontaneous metabolic acidosis (47). It is a condition that has been reported mostly in adults, since its first description in 1959 (48). In these patients, the acidification defect is milder and NH_4_^+^ excretion is greater than in those with complete dRTA (49), a fact that may account for the absence of overt metabolic acidosis. The underlying mechanism that causes the impairment in urinary acidification is not well understood and may be dependent on the associated disorders (47).

Incomplete dRTA has been associated to recurrent calcium kidney stones in adults (50), nephrocalcinosis (50, 51), as well as to osteopenia or osteoporosis (52–54). In these cases, alkali therapy has shown to reduce stone formation and increase bone mass (54, 55). It has also been reported in sickle-cell disease (56), interstitial nephropathies (57) and autoimmune diseases [especially, Sjögren disease (58)].

The diagnosis of incomplete dRTA can be difficult to perform, since, by definition, serum acid-base balance parameters (including HCO_3_^−^) are normal. Incomplete dRTA can be suspected in case of a persistently high UpH, but its confirmation requires the measurement of UpH and UNH_4_^+^ in the same functional tests used to identify complete dRTA (42, 46, 47, 50). However, a low spontaneous UpH is usually considered to rule out an acidification defect without the need for functional tests (41, 47). Although, classically, the threshold value is 5.3 (48), cut-off points between 5.25 and 6.10 have been proposed and used*.* A list of publications on incomplete dRTA screening, along with the threshold values is provided in Table 1.

**Table 1:** Studies that analyze single spot urine pH (UpH) in the context of incomplete dRTA assessment.

Reference (First author. Journal. Year)	Number of patients	Clinical characteristics	Threshold UpH
Tannen. Nephron. 1975 (51)	101	Healthy subjects (75.3%) Recurrent calcium kidney stone formers (16.8%) Nephrocalcinosis (7.9%)	6.0
Norman. J Pediatr. 1978 (59)	22^a^	Healthy subjects (40.9%) Complete dRTA (45.5%) Incomplete dRTA (13.6%)	6.0
Konnak. J Urol. 1982 (60)	5	Recurrent kidney stone formers and/or nephrocalcinosis	5.8
Mateos Anton. Eur Urol. 1984 (61)	50	Recurrent kidney stone formers	6.0
Osther. Scand J Urol Nephrol Suppl. 1988 (62)	40	First kidney stone episode	5.8
Osther. Br J Urol. 1989 (63)	110	Recurrent kidney stone formers	6.0
Gault. Medicine (Baltimore). 1991 (64)	69	Calcium phosphate stones (34.8%) Calcium oxalate stones (43.5%) Healthy subjects (21.7%)	5.25
Chafe. Clin Nephrol. 1994 (30)	110	Recurrent kidney stone formers (87.3%) Healthy subjects (12.7%)	6.10
Weger. Osteoporos Int. 1999 (52)	48	Primary osteoporosis	5.5
Pongchaiyakul Nephrol Dial Transplant. 2004 (65)	361	Healthy subjects in an area of endemic osteoporosis	5.5
Stitchantrakul. J Med Assoc Thai. 2007 (66)	120	Recurrent kidney stone formers (71.7%) Healthy subjects (28.3%)	5.5
Arampatzis. Urol Res. 2012 (67)	150	Male recurrent kidney stone formers	5.8
Shavit. Nephrol Dial Transplant. 2016 (45)	124	Recurrent kidney stone formers and/or nephrocalcinosis	6.0
Dhayat. CJASN. 2017 (29)	170	Recurrent kidney stone formers	5.3
Sromicki. Urolithiasis. 2017 (54)	183	Osteopenia	5.8

Original publications evaluating single spot UpH as a screening tool for incomplete dRTA. The number of patients, its clinical characteristics (conditions that motivate the suspicion of incomplete dRTA), as well as the threshold UpH values (the cut-off points above which an acidification disorder is suspected) are listed. Except when specified, all subjects were adults. In all studies, a confirmatory test of incomplete dRTA was performed (oral NH_4_Cl load and/or furosemide + fludrocortisone test).

^a^ 16 children and 6 adults.

Incomplete dRTA in children has scarcely been reported and its manifestations are not well established. Nevertheless, and, unlike in adults, it has been associated to congenital abnormalities in the kidney and urinary tract (68, 69), asymptomatic hypocitraturia (59), active nutritional rickets (70) and it also has been reported in heterozygous carriers of gene mutations responsible for primary dRTA (71) who did not present spontaneous metabolic acidosis. Furthermore*,* whether it is also a cause of growth retardation in patients with vesicoureteral reflux and posterior ureteral valves (68, 69) without other growth impairment causes, remains an open question. Interestingly, sustained bicarbonate therapy has resulted in growth improvement in some of these cases (72). Therefore, incomplete dRTA evaluation could have important implications in clinical practice for diagnosing causes of growth failure in children, when the cause cannot be identified with routine testing (47, 73).

In addition to the very limited number of published studies on incomplete dRTA in pediatric age, data on UpH values in children are scarce and mostly limited to timed urine samples (74–76). Skinner et al. analyzed in 1996 the first morning UpH in 322 healthy children and found that only one child out of eight had a UpH ≤ 5.4, the median value being 6.0 (77). These results suggest that the utility of fasting UpH in detecting acidification defects is limited, at least when considered in isolation, and point out to the need for more data on pediatric reference UpH values and their relationship with other associated urinary acidification parameters, such as osmolality, electrolytes and, especially, UNH_4_^+^.

## Chronic kidney disease (CKD)

Recent studies have emphasized the importance of measuring UNH_4_^+^ in CKD. An impaired net acid excretion (and, accordingly, low NH_4_^+^ secretion), with the consequent acid accumulation, contributes both to kidney injury (78) and to the pathogenesis of metabolic acidosis, which is a risk factor for progression of renal function deterioration and mortality (79, 80). Indeed, a low 24-hour UNH_4_^+^ level (<20 mmol/day) has been found to be a marker of poor outcome in adult CKD patients without overt metabolic acidosis (80).

Acid accumulation is a well-known risk factor for progression of renal failure and mortality in CKD (80, 81), even when it is insufficient to cause clinically apparent metabolic acidosis, the so-called “eubicarbonatemic” acidosis (78). Acid accumulation in CKD depends, among other factors, on the reduction of GFR, the dietary acid and the integrity of distal acidification mechanisms. Acid-related kidney injury is not limited to patients with reduced GFR and is favored by high-acid diets, such as rich in protein Western diets (78). The identification of eubicarbonatemic acidosis, in order to prescribe dietary interventions or alkali therapy, even in early CKD stages, could decrease acid accumulation and slow CKD progress in these patients (78). Although acid accumulation can be estimated by serum HCO_3_^−^ levels after an oral HCO_3_^−^ load (82), more practical and easier surrogate parameters are needed. In this context, reduced urinary NH_4_^+^ excretion might indicate risk for acid accumulation (78).

These data from adult CKD patients and the clinical implications of eubicarbonatemic acidosis need to be evaluated in children. Congenital kidney and urinary tract abnormalities, which are usually associated to tubulointerstitial injury, constitute a frequent cause of CKD in pediatric patients, especially in younger children (83). In these cases, where tubular function may be impaired already in early CKD stages, there might be a higher proportion of eubicarbonatemic acidosis than in adults, even when GFR is normal. This possibility and the potential clinical utility of UNH_4_^+^ quantification in pediatric CKD patients, need to be explored.

## Final remarks

The identification of a urinary acidification disorder needs a high degree of suspicion and can be difficult, especially when there is not a coexisting metabolic acidosis and serum HCO_3_^−^ is within range, as in incomplete dRTA or eubicarbonatemic acidosis in CKD. Thus, there might be an important number of undiagnosed cases of acidification defects, both in adults and children, which can benefit from early identification and treatment. Since a confirmatory diagnosis requires a functional test, an accurate screening method is necessary. Ideally, this test should be non-invasive and easy to perform, such as the collection of a spot urine sample.

UpH is easy to measure but it cannot be used alone as a screening tool. UNH_4_^+^ represents the most important part of net acid excretion and can provide more information on urinary acidification. Although traditionally not measured in clinical practice due to historical technical difficulties, which have been largely overcome, direct UNH_4_^+^ quantification has gained in importance in the past few years in different conditions.

In fact, UpH and UNH_4_^+^ measurement has shown to be feasible in clinical laboratories nowadays and its applications in clinical practice are starting to be discovered. The assessment of UpH and UNH_4_^+^ should become a part of the evaluation of metabolic acidosis as a simple but informative diagnostic tool. Furthermore, evidence in adult patients also points out to their utility in cases of eubicarbonatemic acidosis and in order to identify incomplete dRTA.

However, there are very few data on UpH and UNH_4_^+^ in pediatric population, so further research is needed to establish reference values in children, either in fasting conditions or in acidosis of nonrenal origin, and to explore the clinical applications of these measurements in metabolic acidosis, in CKD with a reduction of GFR and in incomplete dRTA associated disorders, including growth retardation of unknown cause. The recent availability of morning spot UNH_4_^+^/creatinine ratio reference values in children (38) may clear the path for future studies.
